# Beyond antioxidants: Selenium and skeletal muscle mitochondria

**DOI:** 10.3389/fvets.2022.1011159

**Published:** 2022-12-01

**Authors:** Lauren T. Wesolowski, Pier L. Semanchik, Sarah H. White-Springer

**Affiliations:** Department of Animal Science, Texas A&M University and Texas A&M AgriLife Research, College Station, TX, United States

**Keywords:** selenium, mitochondria, mitochondrial biogenesis, selenoprotein, skeletal muscle

## Abstract

The element, Selenium (Se), has an essential nutritive and biological role as a trace mineral known primarily for its vital antioxidant functions as a constituent of the selenoenzyme, glutathione peroxidase. However, Se also has a much more global biological impact beyond antioxidant function. The objective of this review is to present an overview of prior research on the extra-antioxidant effects of Se with a key focus on skeletal muscle mitochondrial energetics. Cognizance of these additional functions of Se is requisite when formulating and recommending dietary supplementation of Se in humans or animals. Chief amongst its myriad of biological contributions, Se influences mitochondrial capacity and function and, subsequently, muscular health. Dietary Se supplementation has been shown to increase skeletal muscle mitochondrial volume density and within some cell lines, Se treatment increases mitochondrial biogenesis and respiratory capacity. In addition, the selenoproteins H, N, W, and O and deiodinases exhibit varying effects on mitochondrial and/or skeletal muscle function. Selenoprotein H enhances mitochondrial biogenesis whereas selenoproteins N and W appear to influence muscle calcium homeostasis which impacts mitochondrial function. Moreover, selenoprotein O's intramitochondrial residence facilitates Se's redox function. Deiodinases regulate thyroid hormone activation which impacts muscle cell regeneration, metabolism, and reactive oxygen species production. Although the precise relationships between dietary Se and skeletal muscle mitochondria remain unclear, previous research constitutes a firm foundation that portends promising new discoveries by future investigations.

## Introduction

Elemental selenium (Se) was discovered in 1817 by Jöns Jacob Berzelius who happened upon the element when analyzing an unknown impurity present in manufactured sulfuric acid samples (1). He named the compound selenium after the Greek word for moon, *selene*. Upon its discovery, Se was added to the periodic table as atomic number 34 with a molecular weight of 78.971 Da. Since the observance of Se in 1817, numerous reports have revealed its necessity for maintenance of several bodily functions. As such, Se is now considered an essential mineral for humans and animals. Importantly, acute or chronic Se toxicity and deficiency can occur at high and low dietary intake levels, respectively, both of which are accompanied by negative health outcomes. Conversely, balanced dietary Se intake is highly beneficial and well known for its vital antioxidant properties.

Dietary Se can be found in inorganic (e.g., selenite and selenate salts) and organic (e.g., selenomethionine, selenocysteine, and Se-enriched yeast) forms, and the absorption and metabolism of Se differs between the forms. Bioavailability of inorganic Se may be lower than organic Se. Dairy cows fed 3 mg Se/d in the form of Se-yeast had a greater increase in Se concentration in whole blood and had numerically elevated Se concentrations in skeletal muscle, heart, and liver compared to those fed 3 mg Se/d in the form of sodium selenite (2). Rats fed 1, 2, or 4 mg Se/kg diet as selenomethionine had greater Se concentrations in muscle compared to rats fed the same amount of Se as sodium selenite (3). In first parity gilts, Se-yeast supplementation resulted in greater serum Se concentrations at breeding and 90 d post breeding than sodium selenite supplementation, but not at weaning (4). However, Se-yeast supplementation did result in a greater Se content in the loin of the gilts and their progeny immediately after birth and at weanling age (4). Thus, the form of dietary Se may impact the availability of Se and the incorporation of Se into tissues which is important to consider when evaluating results of published works. Regardless of species, Se toxicity must also be considered when determining the form of dietary Se. Organic Se supplementation has been shown to be less toxic than inorganic forms such as groundwater selenate, which has a higher toxicity and should be avoided (5). However, organic Se toxicity is still a potential concern. Some studies suggest intakes >90 μg Se/day (in humans) were associated with greater incidences of diabetes, skin cancer, and prostate cancer (5). More research is encouraged to determine quantitative recommendations of each source of dietary Se to optimize health and performance outcomes.

**Figure 1:**
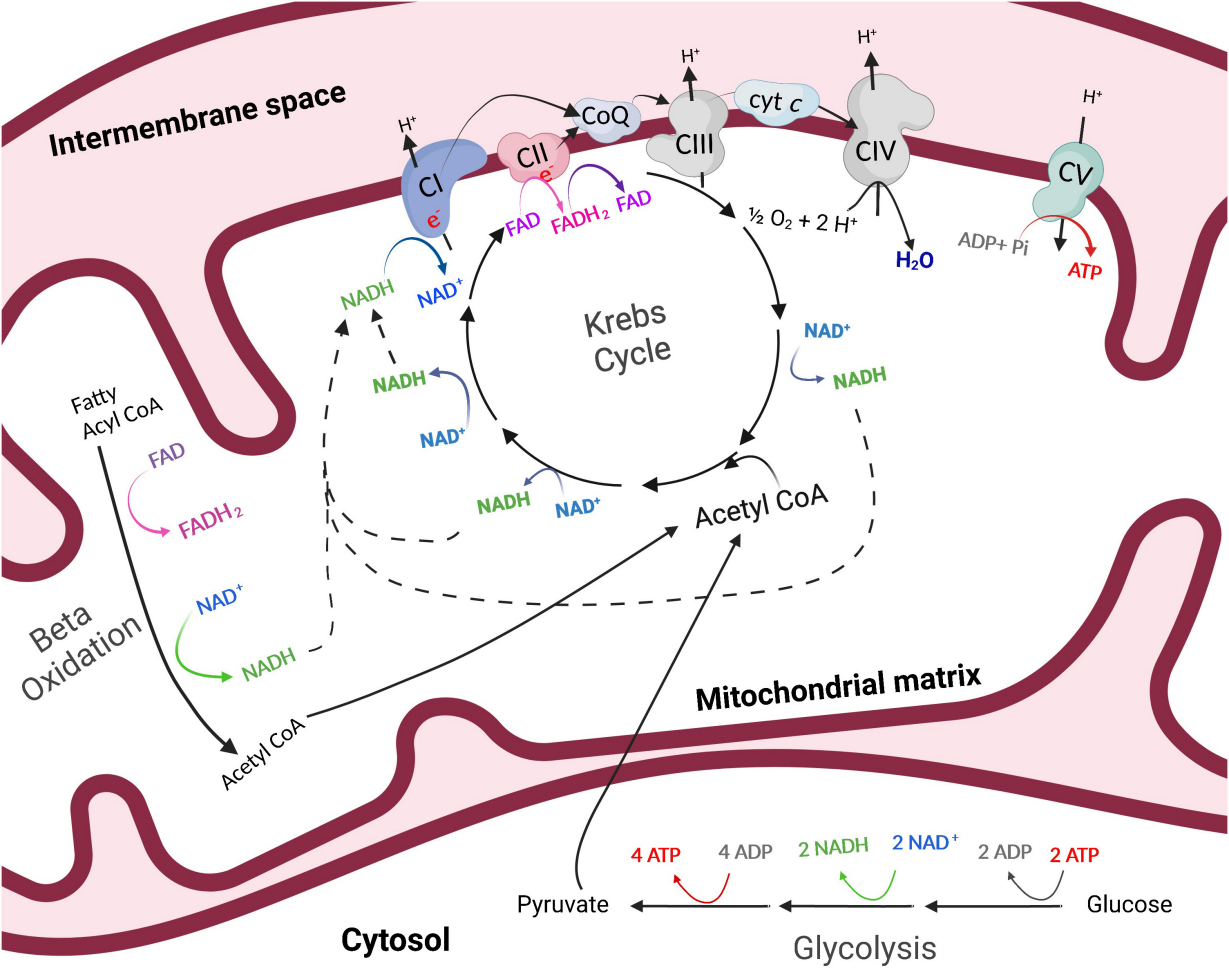
The process of energy production outside of (glycolysis) and within (oxidative phosphorylation) the mitochondria. Through anaerobic glycolysis, glucose is metabolized in the cytosol of the cell to pyruvate. Pyruvate may then be transported into the mitochondria where it is oxidized to acetyl CoA, which enters the Krebs cycle in the mitochondrial matrix. The byproducts of the Krebs cycle, NADH and FADH_2_, provide high-energy electrons to the electron transfer system which resides in the inner mitochondrial membrane. As electrons are transferred, protons are pumped into the intermembrane space of the mitochondria and a proton gradient is generated. Protons then flow through ATP synthase, or complex V (CV) of the electron transfer system, following the chemical gradient to combine with adenosine diphosphate (ADP) and inorganic phosphate (Pi), creating ATP. This process is referred to as oxidative phosphorylation. Created with Biorender.com.

One of Se's most well-known biological roles is as a constituent of several glutathione peroxidase (GPx) isozymes which have an array of functions, including protecting against oxidative stress, minimizing inflammation, and regulating cell death. Examples include GPx1, the most abundant and ubiquitously expressed isozyme which functions to detoxify hydrogen peroxide (H_2_O_2_) to nontoxic water (H_2_O) and GPx4, which has a strong affinity for lipid hydroperoxides and regulates ferroptosis (6). Colloquially known as the “powerhouse of the cell,” mitochondria are the primary source of cellular energy, or adenosine triphosphate (ATP), in the body. During aerobic ATP production, electrons are transferred along a network of protein complexes (I–IV) within the inner mitochondrial membrane known as the electron transfer system (ETS; Figure 1). The movement of electrons along the ETS allows protons to be pumped into the intermembrane space, creating an electrochemical gradient which powers complex V, or ATP synthase. The movement of electrons is not without fault; as a normal byproduct of aerobic ATP production, electrons may leak from the ETS before reaching the final electron acceptor, oxygen. These free electrons may bind with an unpaired oxygen molecule, creating the primary reactive oxygen species (ROS), superoxide (${\text{O}}_{2}^{-}$). Within the mitochondria, leaking electrons resulting in ROS production occurs predominantly at complexes I and III of the ETS in a resting state, though superoxide may also be produced by nicotinamide adenine dinucleotide phosphate (NADPH) oxidase (7). When ROS production overwhelms antioxidant capacity, oxidative stress occurs. The GPx family of enzymes plays a critical role in the prevention of ROS-induced oxidative stress, especially in situations of high stress or energy demand such as during an inflammatory response to injury or pathogens. Thus, both Se and the mitochondria are key biological modulators of skeletal muscle energetics and oxidative stress.

While the antioxidant capabilities of Se have been a popular and integral subject of investigation, there are a vast array of additional biological roles of Se, some which may be extensions of antioxidant activity but others which are unrelated to Se's redox role that may be overlooked. In fact, dietary Se may serve to promote mitochondrial biogenesis, and Se is a component of at least 25 different selenoproteins which have a wide variety of physiological activities. Identification of these other branching properties of Se serves to provide a more comprehensive view of the benefits of dietary Se. Although kidneys have the highest concentration of Se (relative to wet weight), skeletal muscle contains approximately 50% of the body's total Se (8). Therefore, it may be especially important to investigate the role(s) of Se within skeletal muscle. The aim of this review is to characterize the influence of Se on muscle function as it relates to mitochondrial energetics. We intend to demonstrate several lesser-known ways Se is critically intertwined in skeletal muscle mitochondrial health.

## Mitochondrial biogenesis and capacity

Mitochondria are essential organelles for many biological processes due to their role as generators of ATP. The process by which mitochondria produce ATP is known as oxidative phosphorylation (Figure 1). Glucose is metabolized in the cytosol of the cell to pyruvate. In the presence of oxygen, pyruvate is then transported into the mitochondria where it is oxidized to acetyl CoA, which enters the Krebs, or citric acid cycle in the mitochondrial matrix. The byproducts of the Krebs cycle, reduced nicotinamide adenine dinucleotide (NADH) and flavin adenine dinucleotide (FADH_2_), provide high-energy electrons to the ETS which resides in the inner mitochondrial membrane. As electrons are transferred across the complexes of the electron transfer system, protons are pumped into the intermembrane space of the mitochondria and a proton gradient is generated. Protons then flow through ATP synthase along the chemical gradient to combine with adenosine diphosphate (ADP) and inorganic phosphate (Pi), creating ATP (Figure 1). Importantly, mitochondria play pivotal regulatory energetic roles that determine cell growth, metabolism, stress responses, and even cell death.

**Figure 2:**
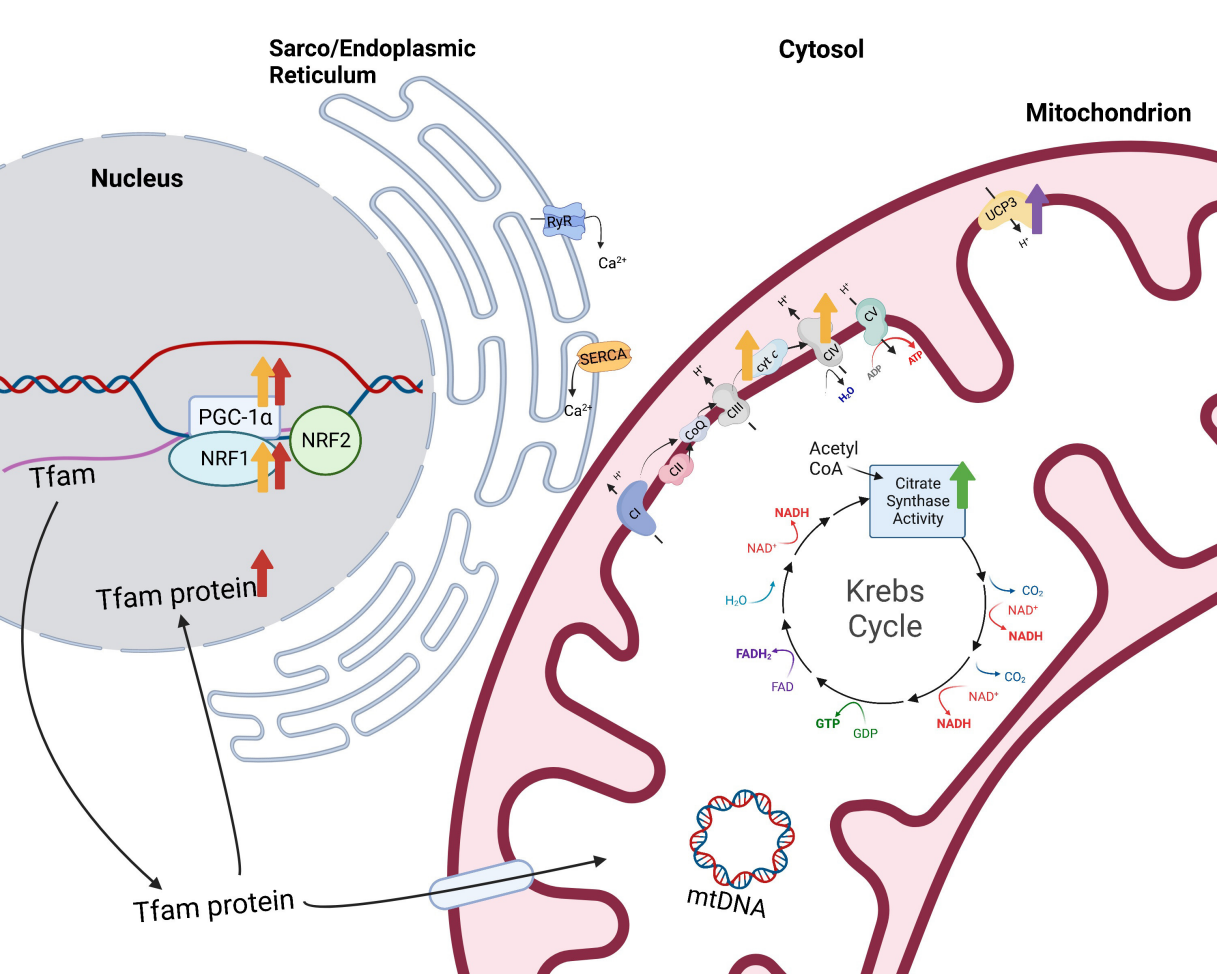
Impacts of selenium supplementation or treatment on mitochondrial function. Citrate synthase activity, a measurement of mitochondrial volume density, is greater (green arrow) in the gluteus medius muscle of horses supplemented 0.3 mg Se/kg DM (9). Additionally, overexpression of the *selenoprotein H* (*SELENOH*) gene in murine hippocampal HT22 neuronal cells resulted in greater levels of mitochondrial biogenesis regulators proliferator-activated receptor-γ coactivator-1 alpha (PGC-1α) and nuclear respiratory factor 1 (NRF1) and mitochondrial transcription factor A (Tfam) within the nuclear fraction [red arrows (10)]. Similarly, sodium selenite-treated HT22 neuronal cells also had greater PGC-1α and NRF1 and greater mitochondrial proteins, cytochrome *c* and cytochrome *c* oxidase IV [yellow arrows (11)]. Finally, type 2 deiodinase (D2) induction in C2C12 cells resulted in increased expression of mitochondrial *uncoupling protein 3* [*UCP3* (12)]. Created with Biorender.com.

A scarce amount of previous research has yielded intriguing results showing that Se supplementation increases skeletal muscle mitochondria. Horses that received 0.3 mg Se/kg dry matter (DM) had greater citrate synthase activity in the gluteus medius muscle at rest compared to horses supplemented with 0.1 mg Se/kg DM [Se supplemented as Se-yeast; Figure 2 (9)]. However, cytochrome *c* oxidase activity was unaffected by Se supplementation. Citrate synthase activity is a commonly used proxy for mitochondrial volume density while cytochrome *c* oxidase activity provides a measure of mitochondrial function (13, 14). Therefore, these results indicate that horses had greater mitochondrial volume density after Se supplementation but no apparent change in mitochondrial function. Somewhat conflictingly, young, male human subjects supplemented 180 μg/d of selenomethionine while endurance training for 10 wk had a lesser increase in vastus lateralis muscle mitochondrial content than non-supplemented individuals in training (15). However, Se supplementation while endurance training caused a greater increase in the size of the individual mitochondria whereas training alone resulted in an increase of the number of mitochondria (15). This suggests a preservation of existing mitochondria due to Se supplementation rather than resynthesis of more small, less mature mitochondria. Importantly, with training, ROS serve as signaling molecules to drive muscle adaptation and, as an antioxidant, Se might limit ROS signals for mitochondrial adaptation. Additionally, the impact of exercise training in combination with Se supplementation may yield varying results dependent upon the intensity of the training protocol. However, in the equine study mentioned above, horses supplemented Se had an increase in skeletal muscle mitochondrial volume density regardless of whether they were untrained or undergoing submaximal training (9). Impacts of dietary Se supplementation on mitochondrial biogenesis *in vivo* during various exercise training regimes warrants further investigation.

It is possible that size and number of mitochondria increase with Se supplementation due to the antioxidant activity of Se protecting cells and organelles from oxidative stress. Supplementation of sodium selenite in mice protected kidneys from cadmium-induced oxidative stress and prevented apoptosis through mitochondrial pathways (16). Specifically, supplementation inhibited mitochondrial membrane potential collapse, cytochrome *c* release, and caspase activation, and prevented a decrease in voltage dependent anion channels, all of which inhibit mitochondrial signaling of cell apoptosis. This signifies that dietary Se might prevent oxidative stress-induced cell death which likely aids in maintaining a larger volume density of mitochondria.

Alternatively, other research presents the possibility that Se induces mitochondrial biogenesis, or the process of increasing mitochondrial cell numbers from pre-existing mitochondria. Overexpression of the *selenoprotein H* (*SELENOH*) gene in murine hippocampal HT22 neuronal cells resulted in greater levels of the “master regulator of mitochondrial biogenesis,” peroxisome proliferator-activated receptor-γ coactivator-1 alpha (PGC-1α), as well as nuclear respiratory factor 1 (NRF1) and mitochondrial transcription factor A (Tfam) within the nuclear fraction compared to vector-transfected cells [Figure 2 (10)]. In addition, mitochondrial biogenesis regulators correlated with greater mitochondrial mass in the *SELENOH* overexpressed cells. Similarly, sodium selenite-treated HT22 neuronal cells also had greater PGC-1α and NRF1 and greater mitochondrial proteins, cytochrome *c* and cytochrome *c* oxidase IV [Figure 2 (11)]. PGC-1α is known as the master regulator of mitochondrial biogenesis because it induces the activation of multiple transcription factors which compensates for the inability of PGC-1α to bind to DNA. These transcription factors include NRF1 and 2, estrogen-related receptors (ERR) α and γ, and peroxisome proliferator-activated receptor α (PPARα). NRF1 and 2 provide transcriptional control of mitochondrial biogenesis associated genes through contact with the promoter of Tfam (17), while ERRα and γ are stimulated to promote ATP uptake and transport across mitochondrial membranes (18), providing energy for expansion of the mitochondrial network. PPARα is co-activated by PGC-1α to regulate fatty acid oxidation and transportation of proteins responsible for linking beta oxidation with mitochondrial biogenesis (19). Additional transcription factors including glucocorticoids, thyroid hormone, and uncoupling proteins play important roles in mitochondrial biogenesis through coactivation by PGC-1α.

**Figure 3:**
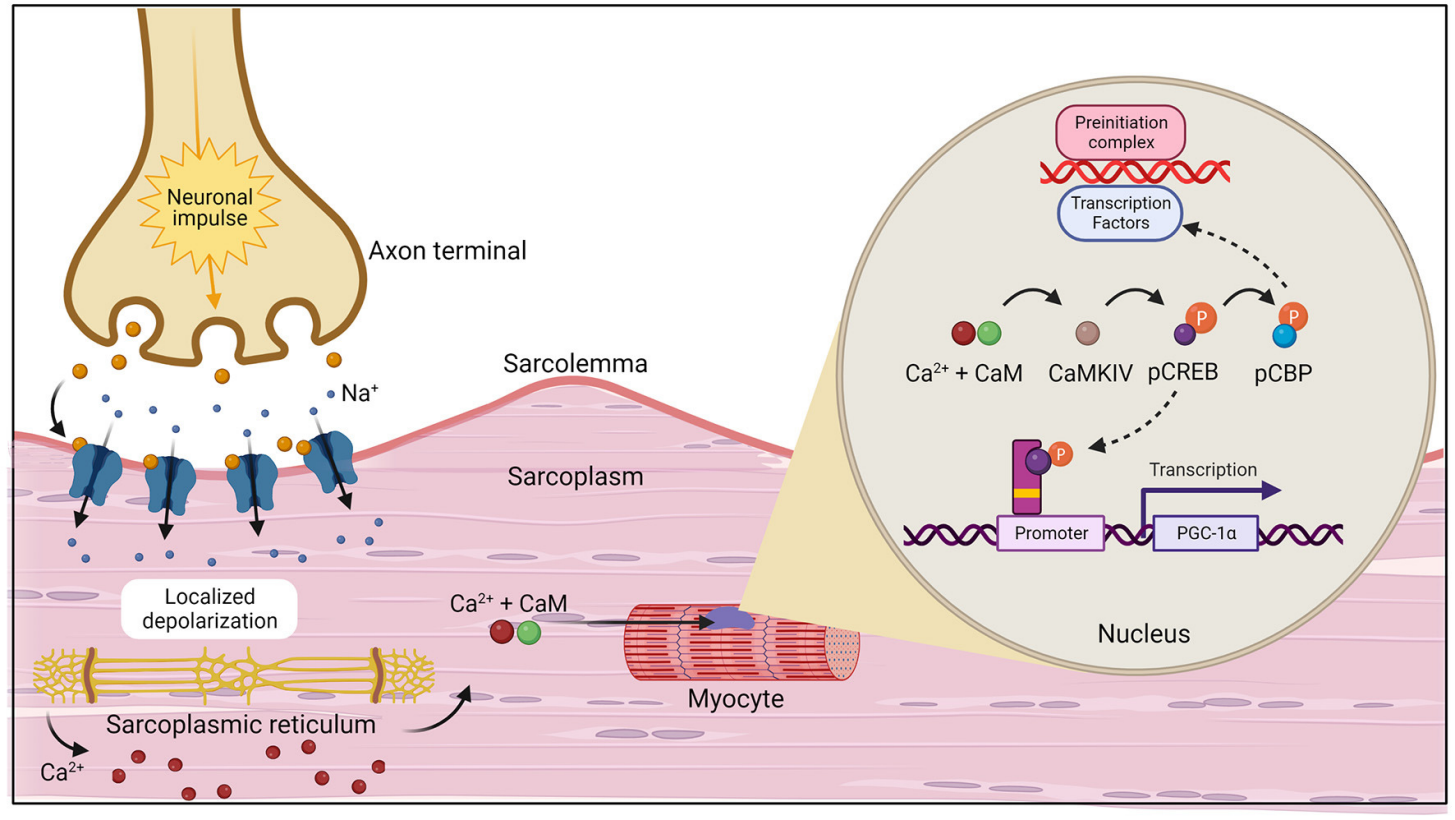
The initiation of muscle contraction followed by the two primary pathways, calcium (Ca^2+^) calmodulin-dependent protein kinase IV (CaMKIV)/calcineurin A (CnA) and cAMP response element-binding protein (CREB) that activate the *PGC-1*α gene which act to increase mitochondrial biogenesis (20). Created with Biorender.com.

During exercise, PGC-1α can be activated through two primary pathways: calcium (Ca^2+^) calmodulin-dependent protein kinase IV (CaMKIV)/calcineurin A (CnA) and cAMP response element-binding protein [CREB; Figure 3 (20)]. CaMKIV and CnA are considered Ca^2+^ sensitive enzymes that respond to elevated intracellular levels of Ca^2+^ which are present during muscle contraction. In the presence of increased levels of Ca^2+^, calmodulin (CaM) will bind and rapidly travel to the nucleus to signal CaMKIV (21). Once activated, CaMKIV will phosphorylate CREB at the Ser133 site (22). Importantly, CaMKIV phosphorylation at this site has been found to be the immediate pathway to creating phosphorylated CREB (pCREB), which occurs directly after potassium depolarization in the contracting muscle. However, a secondary pathway, the mitogen-activated protein kinase (MAPK) pathway, has been demonstrated to be a prolonged kinase of CREB (23, 24). One study examined the time of recruitment for each kinase after a depolarizing stimulus was applied to neurons and found that (1) CaMKIV was predominantly active at 0 to 10 min; (2) both kinases were active at 30 min; and (3) MAPK was the main kinase at 60 min (23). It was concluded that this was the result of the immediate response of calmodulin to lower concentrations of Ca^2+^ when compared to MAPK regulators. Upon formation of the nuclear transcription factor, pCREB, the CREB binding protein (CBP) is recruited and phosphorylated at Ser301 (25). Then, CBP phosphorylation facilitates formation and stabilization of the preinitiation complex through its interaction with a variety of transcription factors (26). Meanwhile, pCREB interacts with a CREB binding site on the promoter of the *PGC-1*α gene to aid in transcription (27). PGC-1α acts as a coactivator as it interacts with NRF1 and NRF2 in addition to ERRs and Tfam, all of which are responsible for transcribing genes that increase electron transport subunits, mitochondrial DNA, and mitochondrial proteins, thus being the primary regulator of mitochondrial biogenesis (28–31).

In addition to increasing mitochondrial biogenesis, Se appears to increase mitochondrial respiratory capacity. When incubated with sodium selenite or selenomethionine, placental tissue and Swan-71, JEG-3, and BeWo trophoblast-like cells exhibited increased respiratory capacity (32). The Se treated cell lines also exhibited greater SELENOH content. Correspondingly, both overexpression of *SELENOH* and sodium selenite treatment in murine hippocampal HT22 neuronal cells resulted in greater oxygen consumption compared to control cells (10, 11). Furthermore, *SELENOH* overexpression prevented a decrease in mitochondrial respiration following UVB-irradiation (10). While these studies were conducted *in vitro*, a recent study in horses investigated the potential for dietary Se to enhance skeletal muscle mitochondrial respiration *in vivo* (33). The results indicated that removing commonly added levels of vitamin E to performance horse diets reduced mitochondrial respiratory capacities but the impairment was rescued by providing horses with 0.3 mg Se/kg DM *via* a proprietary Se yeast blend (33). Importantly, these studies indicate a functional benefit of Se since mitochondrial respiration drives necessary energy production. Further investigation is required to adequately characterize the relationship between Se and mitochondrial biogenesis and capacity, and to determine the mechanisms by which Se enhances mitochondrial respiration.

## Selenoproteins

Many biological effects of Se are mediated by Se-containing proteins, also known as selenoproteins, which generally contain at least one selenocysteine (a Se-containing amino acid and the main biological form of Se) and often serve oxidoreductase functions. This review will discuss four known selenoproteins that may mediate some facet of skeletal muscle mitochondrial function: SELENOH, Selenoprotein N (SELENON), Selenoprotein W (SELENOW), and Selenoprotein O (SELENOO). Additionally, we briefly review the selenoproteins type 2 and type 3 deiodinases (D2 and D3) due to their influence on skeletal muscle regeneration, myogenesis, and metabolism. Previous reports present conflicting results regarding the effect of dietary Se on production of selenoproteins. Broiler chicks supplemented Se *via* sodium selenite had greater expression of *selenoprotein N1, W1*, and *O* in the pectoral muscle compared to non-supplemented chicks (34). However, in rats, only *Sepw1* expression was highly regulated within muscle with sodium selenite and Se-deficiency (35). Therefore, it remains unknown the extent to which se supplementation can impact production and function of the four selenoproteins outlined in the current review. Nevertheless, research does identify multiple integral functions of these selenoproteins within muscle and mitochondria.

### Selenoprotein H

Selenoprotein H was initially identified using bioinformatics methods developed around selenocysteine insertion sequence (SECIS) elements of human genomes (36). The SECIS elements allow selenocysteine to be cotranslationally incorporated into the polypeptide; this allows SECIS elements to be utilized, in part, to identify novel selenoproteins. Since then, early study of SELENOH in zebrafish showed localization in the brain ventricular zone, the branchial arches and pectoral fin buds, and the proliferative zone of the retina. Sequence analysis suggests SELENOH has a redox function and resides in the nucleus (37). Subsequently, *via* Western blot and immunohistochemistry, SELENOH was found in the nucleolar fraction specifically within the nucleoli (37). As detailed in the Mitochondrial biogenesis and capacity section of this review, *SELENOH* expression in neuronal cells appears to play a pivotal role in mitochondrial biogenesis and respiratory capacities. Specifically, overexpression of the *SELENOH* gene in neuronal HT22 cells resulted in greater mitochondrial mass, mitochondrial biogenesis regulators, and oxygen consumption compared to vector-transfected cells (10). Additionally, total and phosphorylated protein kinase A, Akt/protein kinase B, and CREB were significantly increased in SELENOH transfected cells compared to vector transfected neuronal HT22 cells (38). Importantly, CREB senses insufficient energy and may enhance *PGC-1*α transcription to then upregulate mitochondrial biogenesis (20, 39). Protein kinase A and Akt regulate CREB. Alternatively, SELENOH might have antioxidant functions since overexpression of human *SELENOH* in HT22 cells protected against UV-induced cell death by decreasing superoxide levels (40). Selenoprotein H appears to be involved in mitochondrial biogenesis and antioxidant function within neuronal cells. This remains to be investigated within skeletal muscle cells but the potential impact of SELENOH on mitochondrial function is worthy of future consideration.

### Selenoprotein N1

Selenoprotein N was originally characterized by bioinformatics methods in 1999 (41) and was implicated in neuromuscular diseases, specifically rigid spine syndrome, not long after the discovery (42). Many of these neuromuscular diseases are now considered selenoprotein N1 (SELENON also referred to as SEPN1)-related myopathies caused by mutations in the *SELENON* gene which occurs mainly in humans. Selenoprotein N1 is a sarcoplasmic reticulum (SR) transmembrane glycoprotein with a cysteine-selenocysteine active site and an N terminus which is exposed to the cytoplasm. Increased fat mass, decreased global body mass, and increased energy expenditure are noted in *SELENON* knockout mice compared to wild-type mice suggesting SELENON is involved in bioenergetics (43). Mutation of the *SELENON* gene in humans may also result in early truncal hypotonia, neck weakness, progressive scoliosis, and a 12% smaller mean diameter of slow-twitch type I muscle fibers compared to fast-twitch type II fibers (44). Ultimately, these results demonstrate that SELENON is integral in muscle function and metabolism. However, the mechanisms that cause these specific muscular impairments with SELENON loss are still relatively unknown.

**Figure 4:**
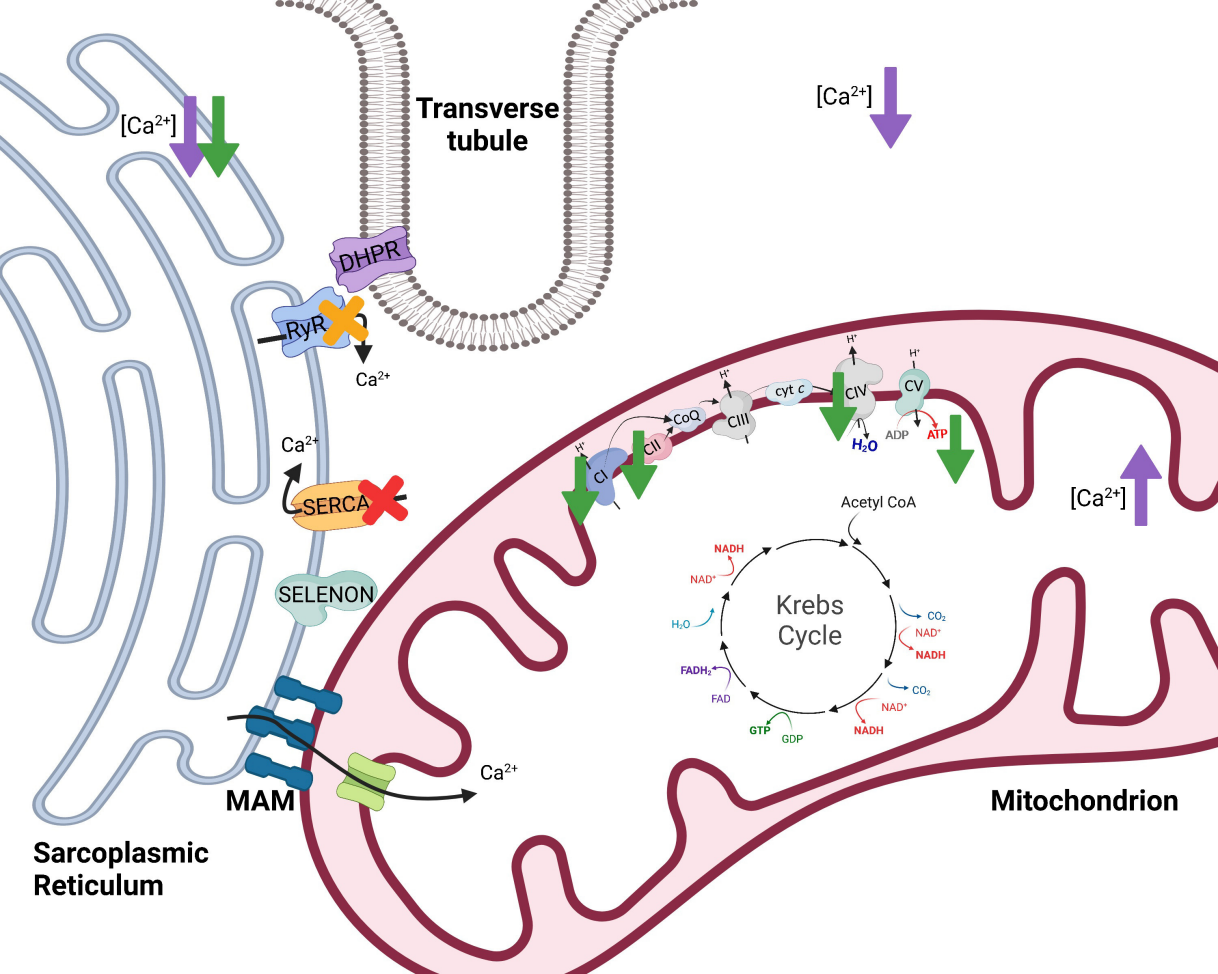
The effects of selenoprotein N1 (SELENON) and selenoprotein W (SELENOW) knockout/deficiency in skeletal muscle. To initiate muscle contraction, an action potential travels down the Transverse tubule (T-tubule) to activate voltage-gated channels known as dihydropyridine receptors (DHPR) triggering the ryanodine receptor (RyR) to release Ca^2+^. When stimulated with 100 mM caffeine, myofibers from SELENON deficient mice had reduced amplitude of Ca^2+^ release which suggests that RyR of the sarcoplasmic reticulum may be impaired with loss of SELENON [yellow X (45)]. Further, SELENON serves to regulate calcium and redox homeostasis by interaction with and activation of the sarco/endoplasmic reticulum calcium transport ATPase (SERCA) 2b [red X (46)]. Cells deficient in SELENON had lower ER Ca^2+^, less Ca^2+^ transport into the mitochondria, reduced ATP content, reduced complex I (CI) activity, and impaired function of complexes II (CII) and IV (CIV) of the electron transfer system [green arrows (43)]. Lastly, SELENOW deficiency in myoblasts resulted in decreased Ca^2+^ in the cytoplasm and sarcoplasmic reticulum but increased Ca^2+^ levels in mitochondria [purple arrows (47)]. Created with Biorender.com.

Selenoprotein N1 serves to regulate Ca^2+^ and redox homeostasis by interaction with and activation of the sarco/endoplasmic reticulum Ca^2+^ transport ATPase 2b [SERCA2b; Figure 4 (46, 48)]. Importantly, SERCA pumps dictate the resting cytosolic Ca^2+^ concentrations because SERCA pumps have a high affinity for removal of Ca^2+^ from the cytosol. Within skeletal muscle, release of Ca^2+^ from the SR and subsequent binding of Ca^2+^ to troponin C is required to induce muscle contraction. However, removal of Ca^2+^ from the cytosol is of equal importance since it functions to stop contraction when necessary. Thus, impairment or loss of SELENON could influence SERCA mediated Ca^2+^ homeostasis within muscles, likely impairing the re-uptake of Ca^2+^ into the SR and limiting muscles from returning to a resting, non-contracted state. Conversely, when stimulated with 100 mM caffeine, flexor digitorum brevis myofibers from SELENON deficient mice had reduced amplitude of Ca^2+^ release compared to wild-type mice which suggests that the ryanodine receptor of the SR may be impaired with loss of SELENON (45). The ryanodine receptor is involved in release of Ca^2+^ from the SR so, although it is evident that SELENON loss results in impairment of Ca^2+^ homeostasis, the origin of this defect remains unclear and may involve both the release (ryanodine receptor) and re-uptake (SERCA) of Ca^2+^.

The actions of SELENON are also thought to influence mitochondrial function because SELENON is localized near mitochondria-associated membranes (MAMs) of the SR. Along with the SR, mitochondria assist in maintaining Ca^2+^ homeostasis. The MAMs serve several functions, one of which being that the SR and mitochondria can transfer Ca^2+^ at MAMs [Figure 4 (49)]. Within skeletal muscle, SR and mitochondria locally transfer Ca^2+^ in response to caffeine stimulation (50). Physiological concentrations of Ca^2+^ regulate multiple mitochondrial enzymes (51–54) and Ca^2+^ can increase maximum velocity of oxidative phosphorylation by activating many aspects of the oxidative phosphorylation pathway (55). Cells deficient in SELENON had lower ER Ca^2+^ and less Ca^2+^ transport into the mitochondria (43) potentially influencing mitochondrial oxidative phosphorylation.

Absence of SELENON in mice resulted in decreased mitochondrial respiratory capacities in the tibialis anterior, diaphragm, and quadriceps muscles compared to wild-type mice (43). SELENON-devoid cells also had reduced ATP content, slightly decreased mitochondrial membrane potential, reduced complex I activity, and impaired function of complexes II and IV of the ETS [Figure 4 (43)]. In support of this, *SELENON* knockout mice have lower blood glucose and faster muscle and liver glycogen depletion in response to exercise (43) potentially indicating an increased reliance on anaerobic glycolytic metabolism. Anaerobic glycolysis is a less efficient form of energy production and occurs outside of the mitochondria while oxidative metabolism is a more efficient form of energy production and is carried out within the mitochondria (Figure 1). Blunted mitochondrial function due to lack of SELENON could induce a shift toward increased use of anaerobic metabolism to produce necessary cellular energy. Some human patients with SELENON-related myopathy have low body mass index as well as abnormal glucose metabolism (56) which might be a functional result of altered SR regulation and mitochondrial metabolism. Ultimately, SELENON is integral in maintaining Ca^2+^ homeostasis but further study is required to elucidate the full influence of SELENON on mitochondrial function and metabolism.

### Selenoprotein W

One myopathy which is caused by Se deficiency is white muscle disease within young livestock animals. The disease was discovered to be linked to Se deficiency in 1958 (57) which then led to investigation of selenoproteins within muscle and prompted the identification of SELENOW (58, 59). Similar to many of the selenoproteins, SELENOW is highly conserved in mammals. Specifically, SELENOW has been studied in humans, monkeys, rats, mice, sheep, and pigs. Selenoprotein W accumulation is highest within skeletal muscle, heart, and brain but this does not correlate with Se concentrations which are highest within kidneys and lowest in skeletal muscle (60). The exact function(s) of SELENOW remains unknown but SELENOW in rats can bind to glutathione (61) which suggests a redox function. On the other hand, the recombinant form of rat mutant SELENOW in *E. coli* was glutathione bound under anaerobic conditions but not under aerobic conditions indicating SELENOW may exist with or without glutathione (62). There is high expression of SELENOW in proliferating myoblasts but there is minimal expression in differentiated myotubes indicating a potential importance in muscle differentiation (63). Furthermore, SELENOW might regulate Ca^2+^ homeostasis since SELENOW deficiency results in altered Ca^2+^ accumulation and expression of Ca^2+^ channels (47). It is still unclear which functions SELENOW serves, and it is possible that, under different biological conditions, SELENOW has differing functions.

Animals with the peracute form of white muscle disease may present with dysrhythmias, exhaustion, and cardiovascular collapse while animals with the subacute form may have dysphagia, muscular weakness, muscular pain, and more (64). Since these clinical signs are accompanied by low serum antioxidants, GPx and vitamin E, it has been proposed that white muscle disease is induced by ROS and oxidative stress. However, decreased SELENOW has also been observed in the muscle of animals with white muscle disease. In addition to decreased SELENOW, animals with white muscle disease can have reduced uptake of Ca^2+^ into the SR (65) signifying a potential role of both SELENOW and irregular Ca^2+^ homeostasis in the disease. Total Ca^2+^ levels in the muscle were decreased in broiler chickens fed a Se-deficient diet (47). Within myoblasts, a specific SELENOW deficiency also resulted in decreased Ca^2+^ in the cytoplasm and SR but increased Ca^2+^ levels in mitochondria [Figure 4 (47)]. Mitochondria also exhibited swelling, dilation, disruption of cristae, and decreased mitochondrial membrane potential with SELENOW deficiency. The SELENOW deficient myoblasts had decreased expression of SERCA and ryanodine receptors 1 and 3 (47). Thus, deficiency of SELENOW likely causes impaired ability to release and reuptake Ca^2+^ from the SR which may induce altered Ca^2+^ concentrations and mitochondrial deformities. Ultimately, however, oxidative stress also occurs with Se and SELENOW deficiencies, and the mechanism behind the clinical outcomes of white muscle disease and Se deficiency might be multifactorial. Further investigation could aid in elucidating the exact role of SELENOW in muscle and its influence on mitochondrial function.

### Selenoprotein O

Similar to the previously mentioned selenoproteins, SELENOO was originally identified as a selenoprotein using bioinformatics (36). The exact functions of SELENOO, like several other selenoproteins, remain relatively unknown. Presence of a mitochondrial leader sequence in SELENOO, and the localization of SELENOO in the mitochondrial fraction of human embryonic kidney 293T cells indicate that SELENOO likely resides in the mitochondria (66). Additionally, SELENOO might have a redox reaction with another protein through its selenocysteine residue. Selenoprotein O in human embryonic kidney 293T cells is reversibly oxidized when treated with H_2_O_2_ which also supports the theory of a redox function. It is possible the redox function involves kinase action and regulation of signaling cascades (66). Structural and functional analysis of SELENOO using bioinformatics suggests a three-dimensional fold similar to protein kinases (67). Further, SELENOO transfers adenosine monophosphate (AMP) from ATP to protein substrates (AMPlaytion) that are involved in redox homeostasis (68). Interestingly, SELENOO appears to reside within the mitochondria, but future research is warranted to investigate the significance of its localization and proposed functions.

### Deiodinases

Thyroid hormone (TH) serves several critical regulatory functions, including the regulation of satellite cells, or muscle stem cells. Selenoenzymes D2 and D3 are present in skeletal muscle, and their expression influences intracellular TH levels consequently impacting stem cell proliferation and differentiation. The activation, proliferation, fusion, and differentiation of normally quiescent satellite cells is necessary for muscle fiber regeneration following injury. Type 3 deiodinase deactivates thyroxine (T4) and 3,5,3′-triiodothyronine (T3). Specifically, D3 is highly expressed in proliferating myoblasts but lowly expressed in differentiated myoblasts (69). High expression of D3 limits activated TH levels which likely allows for normal satellite cell proliferation and prevention of TH-induced cell apoptosis. Depletion of D3 during *in vivo* muscle regeneration disrupted the regeneration process due to rapid cell apoptosis (69). Conversely, D2 is involved in regulation of muscle fiber regeneration *via* promotion of the differentiation stage. Type 2 deiodinase converts the pro-hormone T4 into T3, the active hormone. *In vitro* and *in vivo*, the absence of D2 limits intracellular T3 concentrations which prevents muscle cell differentiation (70). Thus, both D3 and D2 serve to regulate different stages of muscle satellite cell regeneration [for a full review of D2 and D3 functions, see (71–73)].

The regulation of conversion of T4 to T3 by D2 may impact mitochondria, as well. Low plasma levels of T3, T4, or thyroid stimulating hormone (TSH) stimulate the hypothalamus to release thyrotropin-releasing hormone which, in turn, stimulates the anterior pituitary to release TSH, promoting T4 release from the thyroid. On the contrary, high plasma levels of T3, T4, and TSH serve as negative feedback signals to both the hypothalamus and the anterior pituitary. Knockout of the *Dio2* gene resulted in elevated serum TSH levels due to the lack of negative feedback from elevated T4 (12). This could be critical since cultured equine skeletal muscle fibers treated with 10 mIU TSH showed increased mitochondrial oxidative phosphorylation capacity (74). Further, T3 injections increased PGC-1α protein content in rat skeletal muscle (75). Therefore, increases in D2 may serve to downregulate TSH production and, consequently, mitochondrial biogenesis and/or capacity. This is supported by data showing that inducing D2 enzyme activity and protein expression increased intracellular TH action prompting a net shift from oxidative to glycolytic metabolism. The shift in metabolism was also associated with changes in myosin heavy chain (MyHC) expression toward faster, more glycolytic isoforms, MyHC IIa and IIb. However, the shift to glycolytic metabolism did not alter total ATP production. Interestingly, D2 appeared to support multiple antioxidant functions, including upregulation of expression of the antioxidant, *superoxide dismutase 2* (*SOD2*), as well as mitochondrial *uncoupling protein 3* (*UCP3*; Figure 2) which may facilitate movement of protons back into the mitochondrial matrix to bind unpaired electrons. In support, D2 induction reduced mitochondrial ROS levels which promoted cell differentiation (76). Through mediating TH activity, D2 and D3 influence both muscle metabolism and satellite cell proliferation and differentiation, and D2 may impact mitochondrial capacities through TSH regulation.

## Future directions

Current literature suggests a close link between skeletal muscle mitochondria and Se. It appears that several of the impacts of Se on mitochondrial function may be extensions of the antioxidant properties of Se while other effects of Se stem from the more elusive functions of selenoproteins. Specifically, Se increases mitochondrial biogenesis and function, but this could be, in part, due to antioxidant protection. On the other hand, selenoproteins like SELENON and SELENOW may regulate muscular Ca^2+^ signaling and SELENOO resides in the mitochondria and could serve redox functions. However, the precise mechanisms of the Se-mitochondria relationship remain unknown, therefore it is difficult to surmise specific supplementation recommendations to optimize function of skeletal muscle mitochondria. Additionally, much of the literature reviewed in the present manuscript has investigated the impacts of Se or selenoproteins on mitochondria in cell or rodent models with a few other papers focusing on horses (9, 33), humans (15), or chickens (47). Thus, in humans, livestock, and companion animals there is currently no conclusive understanding of how Se supplementation influences skeletal muscle mitochondrial function beyond antioxidants. To address this, there are several methods and technologies which should be utilized to investigate different aspects of mitochondrial function. Mitochondrial volume density can be measured *via* transmission electron microscopy, cardiolipin and mitochondrial DNA content, citrate synthase activity, and more. Additionally, mitochondrial respiratory capacity can be assessed by respirometry, the measurement of oxygen consumption which occurs during oxidative phosphorylation. There are several systems used to perform respirometry including Clark electrode systems, the Seahorse, and the Oxygraph 2k [for a full review, see (77)]. Future studies may look toward implementation of these measures of mitochondrial volume density and respirometry to assess skeletal muscle mitochondrial function following supplementation of Se under different conditions. Some specific areas of interest include the impacts of Se supplementation on mitochondria during growth and development, exercise training, and stress. Importantly, further investigation of the effect of Se supplementation on skeletal muscle mitochondria could provide prevalent information for future Se treatment to optimize mitochondrial function in various species. Enhancement of mitochondrial energy production could provide numerous benefits such as preventing fatigue within exercising muscle (78).

## Conclusion

There is minimal current literature on the effects of Se on skeletal muscle mitochondria beyond its antioxidant role(s). However, several studies provide multiple theories and potential avenues for Se influence on mitochondria. Selenium may increase mitochondrial biogenesis, but the magnitude of this impact could differ by tissue, exercise intensity and/or duration, and source of dietary Se. Selenoproteins have a wide range of functions which are not fully elucidated but could influence mitochondrial function. Some of these potential functions of selenoproteins include promoting satellite cell differentiation, mitochondrial biogenesis, redox functions, and regulating Ca^2+^ homeostasis which influences mitochondrial capacity. This review had the specific focus of outlining the non-antioxidant influence of Se on muscle function as it relates to mitochondrial energetics. Therefore, the authors acknowledge that, due to the scope of this review, there is additional existing literature on the impacts of Se that was regretfully not included in the current paper. Nevertheless, the compelling literature covered in the current review demonstrates several ways in which Se impacts skeletal muscle health and mitochondrial function. These results highlight the importance of future studies to classify the exact mechanisms behind the influence of Se on mitochondria because there is much more to Se beyond antioxidants.

## Author contributions

SW-S conceived the subject of the review and provided direction on the content of the manuscript. LW, PS, and SW-S wrote the manuscript. All authors approved the final version of the manuscript.

## Funding

Publication fees were provided by the Texas A&M Department of Animal Science.

## Conflict of interest

The authors declare that the research was conducted in the absence of any commercial or financial relationships that could be construed as a potential conflict of interest.

## Publisher's note

All claims expressed in this article are solely those of the authors and do not necessarily represent those of their affiliated organizations, or those of the publisher, the editors and the reviewers. Any product that may be evaluated in this article, or claim that may be made by its manufacturer, is not guaranteed or endorsed by the publisher.

## Abbreviations

ADP: Adenosine diphosphate
AMP: Adenosine monophosphate
ATP: Adenosine triphosphate
CaM: Calmodulin
CaMKIV: Calmodulin-dependent protein kinase IV
CnA: Calcineurin A
CREB: cAMP response element-binding protein
D2: Type 2 deiodinase
D3: Type 3 deiodinase
DHPR: Dihydropyridine receptor
DM: Dry matter
ER: Endoplasmic reticulum
ERR: Estrogen-related receptors
ETS: Electron transfer system
FADH_2_: Flavin adenine dinucleotide
GPx: Glutathione peroxidase
MAM: Mitochondria-associated membrane
MAPK: Mitogen-activated protein kinase
MyHC: Myosin heavy chain
NADH: Reduced nicotinamide adenine dinucleotide
NADPH: Nicotinamide adenine dinucleotide phosphate
NRF: Nuclear respiratory factor
PGC-1α: Proliferator-activated receptor-γ coactivator-1α
Pi: Inorganic phosphate
PPARα: Peroxisome proliferator-activated receptor α
ROS: Reactive oxygen species
Se: Selenium
SELENOH: Selenoprotein H
SELENON: Selenoprotein N
SELENOO: Selenoprotein O
SELENOW: Selenoprotein W
SERCA: Sarco/endoplasmic reticulum Ca^2+^ transport ATPase
SOD2: Superoxide dismutase 2
SR: Sarcoplasmic reticulum
T3, 3, 5: 3′-triiodothyronine
T4: Thyroxine
Tfam: Mitochondrial transcription factor A
TH: Thyroid hormone
TSH: Thyroid stimulating hormone
UCP3: Uncoupling protein 3.
